# CK19 mRNA in blood can predict non-sentinel lymph node metastasis in breast cancer

**DOI:** 10.18632/oncotarget.8851

**Published:** 2016-04-20

**Authors:** Xing-Fei Yu, Hong-Jian Yang, Lei Lei, Chen Wang, Jian Huang

**Affiliations:** ^1^ Department of Surgical Oncology, Second Affiliated Hospital, Zhejiang University School of Medicine, Hangzhou, 310009, P.R.China; ^2^ Department of Breast Tumor Surgery, Zhejiang Cancer Hospital, Banshan Bridge, Hangzhou, Zhejiang Province, 310022, P.R.China; ^3^ Department of Breast Medical Oncology, Zhejiang Cancer Hospital, Banshan Bridge, Hangzhou, Zhejiang Province, 310022, P.R.China

**Keywords:** breast cancer, CK19, non-sentinel lymph node, metastasis, biomarker

## Abstract

Reverse-transcription polymerase chain reaction (RT-PCR) is used to detect CK19 mRNA in sentinel lymph node biopsy (SLNB) tissues from breast cancer patients. We examined whether CK19 mRNA in peripheral blood is predictive of non-sentinel lymph node (nSLN) metastasis. Breast cancer cases diagnosed with clinical stage cT1–3cN0 and registered in our medical biobank were identified retrospectively. This study then included 120 breast cancer cases treated at Zhejiang Cancer Hospital from Aug 2014 to Aug 2015, including 60 SLN-positive and 60 SLN-negative cases. CK19 mRNA levels in peripheral blood samples were assessed using RT-PCR prior to tumor removal. During surgery, if SLNB tissue showed evidence of metastasis, axillary lymph node dissection (ALND) was performed. No ALND was performed if SLNB and nSLN tissues were both negative for metastasis. CK19 expression was higher in nSLN-positive patients than in nSLN-negative patients (*p* < 0.05). Logistic regression indicated that lymphatic vessel invasion and CK19 levels were predictive of nSLN status (*p* < 0.05). The area under the ROC curve for CK19 was 0.878 (*p* < 0.05). We conclude that high CK19 levels in peripheral blood may independently predict nSLN metastasis in breast cancer patients.

## INTRODUCTION

Breast cancer is the most frequently diagnosed cancer and the leading cause of cancer death among females worldwide, with an estimated 1.7 million cases annually [1]. Current practice guidelines recommend complete axillary lymph node dissection (ALND) only in sentinel lymph node (SLN)-positive breast cancer patients. Nonetheless, in more than half of these patients, histologic evaluation of all removed axillary tissue reveals that the SLN was the only lymph node showing evidence of metastasis [2–4]. nSLN metastasis risk depends on various factors, including primary tumor size and SLN metastasis, ratio of number of positive SLNs to all removed SLNs and extracapsular extension of positive SLNs [8–10]. Unfortunately, no single characteristic can independently identify patients with very low risk for additional nSLN metastasis for which ALND could safely be omitted [11]. However, recent years have seen a steep increase in the development of models predicting the probability of non-SLN (nSLN) metastasis in SLN-positive breast cancer patients [6–7]. Such predictive models may provide evidence-based input for decision-making by estimating an individual patient's risks and benefits with regards to specific therapeutic options, such as ALND [5].

The intermediate filament protein cytokeratin 19 (CK19) is a specific epithelial marker [12] that is normally not expressed in lymphoid or hematopoietic tissues [13, 14]. CK19 mRNA detected by reverse-transcription polymerase chain reaction (RT-PCR) has been used as an index of disseminated tumor cells in blood, bone marrow and lymph nodes in breast cancer patients. Recently, a new semi-automated molecular method for rapid intra-operative diagnosis of lymph node metastases in cancer patients was developed using one step nucleic acid amplification (OSNA). This technique has been studied in patients with gastric [15], colorectal [16] and breast cancer [17]. However, CK19 mRNA testing in lymph node or cancer tissue still faces challenges. Testing is intra-operative, and is not valuable for predicting lymph node status prior to surgery. Additionally, while OSNA is helpful in differentiating pN0 or pN+, its utility for differentiating N1/N2/N3 is still unknown.

Oloomi, *et al.* reported that most CK19 mRNAs detected in blood and tissue of cancer patients were non-coding RNAs [18]. Our study also primarily detected small non-coding CK19 RNA fragments in patient samples. Harriette, *et al.* noted the significant association of blood CK19 positivity with distant metastatic versus both node-negative and node-positive breast cancer, but not with any other histopathological parameters examined [19]. The present retrospective study measured pre-surgery CK19 mRNA levels in the peripheral blood of breast cancer patients to evaluate the significance of this marker in predicting nSLN metastasis.

## RESULTS

### Patient clinical and pathological parameters and CK19 mRNA levels

This study analyzed 120 breast cancer cases treated at Zhejiang Cancer Hospital between Aug 2014 and Aug 2015. The average number of SLNs was 2.82 per patient. There were 60 SLN positive and 60 SLN negative cases; 82 cases were nSLN negative and 38 were nSLN positive (Table 1). There were no significant differences in age or expression of human epidermal growth factor receptor-2 (HER2), estrogen receptor (ER) and progesterone receptor (PR) between nSLN negative and positive patients. Patients with positive nSLNs had larger (*p* < 0.05), higher-grade tumors (*p* < 0.05) and increased lymphatic vessel invasion (*p* < 0.05) than patients with negative nSLNs. CK19 mRNA was detected more frequently in nSLN positive patient blood than in nSLN negative blood (*p* < 0.05) (Table 2). Ignoring SLN status, the sensitivity of CK19 for predicting nSLN status was 76.32% and specificity was 96.30%.

**Table 1:** SLN and nSLN status in all patients

		SLN		Total
		Positive	Negative	
nSLN	Positive	38	-	38
	Negative	22	60	82
Total		60	60	120

**Table 2:** Clinical features and CK19 mRNA levels in all patients, regardless of SLN status

		nSLN Negative (*N* = 82)	nSLN Positive (*N* = 38)	*P*-value
Age (years)		48.12 ± 7.67	49.58 ± 7.78	0.892
Tumor size (mm)		25.32 ± 11.99	36.53 ± 19.10	0.000*
Total umber of positive lymph nodes		0.39 ± 0.70	11.95 ± 8.42	0.000*
Grade	1	12	2	0.022*
	2	54	20	
	3	16	16	
lymphatic vessel invasion	No	48	2	0.000*
	Yes	34	36	
HER2	Positive	18	12	0.257
	Negative	20	8	
ER	Positive	62	30	0.688
	Negative	20	8	
PR	Positive	56	28	0.549
	Negative	26	10	
CK19	Postive	4	29	0.000*
	Negative	78	9	
Copies of CK19		174.70 ± 337.39	1136.00 ± 602.03	0.000*

Abbreviations: HER2, human epidermal growth factor receptor-2; ER, estrogen receptor; PR, progesterone receptor.

* χ-test or *t*-test, *p* < 0.05.

### SLN positive patient clinical and pathological parameters and CK19 mRNA levels

There were 22 SLN-positive patients cases with negative nSLNs and 38 cases with positive nSLNs. There were no significant differences in age, tumor size, tumor grade, lymphatic vessel invasion, or HER2, ER and PR levels between nSLN negative and positive patients. Patients with positive SLNs and negative nSLNs had no positive CK19 expression in blood. In SLN-positive patients, CK19 expression was higher in nSLN positive patients compared to nSLN negative patients (*p* < 0.05) (Table 3). For SLN-positive patients, the sensitivity of CK19 for predicting nSLN status was 76.32% and specificity was 100.00%.

**Table 3:** Clinical features and CK19 mRNA levels in SLN(+) patients

		nSLN Negative (*N* = 22)	nSLN Positive (*N* = 38)	*P*-value
Age(years)		48.00 ± 5.92	49.58 ± 7.78	0.250
Menopause	No	14	24	0.970
	Yes	8	14	
Tumor size(mm)		32.27 ± 15.78	36.53 ± 19.10	0.307
Total umber of positive lymph nodes		1.45 ± 0.51	11.95 ± 8.42	0.000*
Grade	1	2	2	0.487
	2	14	20	
	3	6	16	
lymphatic vessel invasion	No	4	2	0.108
	Yes	18	36	
HER2	Positive	8	12	0.705
	Negative	14	8	
ER	Positive	16	30	0.583
	Negative	6	8	
PR	Positive	16	28	0.936
	Negative	6	10	
CK19	Postive	0	29	0.000*#
	Negative	22	9	
Copies of CK19		120.97 ± 198.16	1136.00 ± 602.03	0.000*

Abbreviations: HER2, human epidermal growth factor receptor-2; ER, estrogen receptor; PR, progesterone receptor.

* χ-test or *t*-test, *p* < 0.05.

# Fisher's exact test, *p* < 0.05.

### Logistic regression of factors to predict nSLN status

All clinical features, including age, tumor size, tumor grade, lymphatic vessel invasion, and HER2, ER, PR and CK19 mRNA levels were included in binary logistic regression analysis. Lymphatic vessel invasion and CK19 levels correlated with positive nSLN (*p* < 0.05, Table 4). PR level also showed a weak relationship (*p* = 0.049) with nSLN status.

**Table 4:** Logistic regression of factors nSLN predicting status (enter method)

	B	Wald	*P*-value	Exp (B)	95% CI of Exp (B)	
Age	0.059	1.857	0.173	1.061	0.975	1.154
Tumor size	0.036	1.667	0.197	1.037	0.981	1.095
HER2 (P*)	−1.548	3.005	0.083	0.213	0.037	1.224
ER (P)	−1.859	2.900	0.089	0.156	0.018	1.324
PR (P)	2.087	3.868	0.049	8.057	1.007	64.451
Grade (1)	−2.234	3.421	0.064	0.107	0.010	1.143
Grade (2)	−1.257	1.955	0.162	0.285	0.049	1.657
lymphatic vessel invasion (P)	−3.845	20.063	0.000	0.021	0.004	0.115
CK19	1.715	4.760	0.029	5.554	1.190	25.914

Abbreviations: HER2, human epidermal growth factor receptor-2; ER, estrogen receptor; PR, progesterone receptor.

* P for Positive.

### CK19 mRNA ROC curve for predicting nSLN status

**Figure 1:**
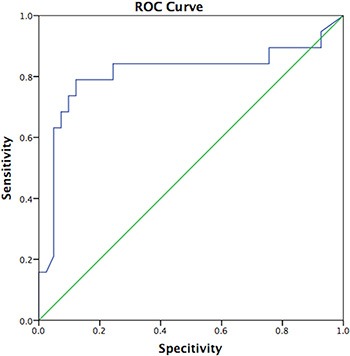
CK19 copy number and nSLN status The area under the ROC curve was 0.878. (*p* < 0.05, 95% CI was 0.793~0.963).

We used quantitative CK19 copy number data for ROC curve analysis. The area under the ROC curve was 0.878, and the 95% CI was 0.793~0.963, suggesting that CK19 level can predict nSLN status pre-surgery (*p* < 0.05, Figure 1).

## DISCUSSION

Current guidelines recommend ALND in SLN-positive breast cancer patients [22]. However, in more than half of these patients, the SLN is the only lymph node positive for metastasis [23–25], and many patients are currently subjected to unnecessary surgery. Models to predict nSLN metastasis in SLN-positive breast cancer patients can potentially contribute to clinical decision-making concerning axillary surgery [7], although improved models are still needed. A recent study of multiple models predicting nSLN involvement in SLN-positive breast cancer patients showed unacceptably high variability between the models [26]. Reynders, *et al.* reported a new method for predicting nSLN metastases in SLN-positive patients that had an area under the curve (AUC) of 0.75, although further validation will be needed prior to use [27].

CK19, a characteristic intermediate filament of epithelial cells and their malignant counterparts, is one of the most frequently studied markers for micro-metastasis. CK19 mRNA expression in peripheral blood has been associated with poor patient outcome [28, 29], and CK19 has been detected in bone marrow and tumor cells from breast cancer patients via immunoassay [30]. OSNA-CK19 has proven useful to detect sentinel lymph node tumor involvement in breast cancer patients [31]. Still, this technique must be performed during SLNB, and is not informative prior to surgery.

While additional validation studies are needed, the present study showed that CK19 can be detected in peripheral blood samples of breast cancer patients, and can predict nSLN status before surgery. Further, CK19 copy number was strongly correlated with the number of metastasis-positive LNs. The inclusion of this tumor marker within already-existing predictive models, which are currently primarily based on clinicopathologic data, would enhance the predictive accuracy of these models in determining LN status in breast cancer patients even before surgery.

## MATERIALS AND METHODS

### Patient eligibility

This study included 120 breast cancer cases treated at Zhejiang Cancer Hospital from Aug 2014 to Aug 2015. Patients meeting all of the following requirements were eligible for enrollment: (1) diagnosis of invasive breast cancer confirmed by histology; (2) clinical stage cT1–3 and cN0 (AJCC, 7th), preparing for SLNB; (3) untreated before diagnosis; (4) voluntary written informed consent obtained; (5) registered in our medical biobank with a peripheral blood sample collected. Criteria for patient withdrawal from the study included: pregnancy, breast-feeding or non-cancer-related illnesses that precluded surgical tumor resection.

### Peripheral blood sample collection

Patient peripheral blood samples were collected one day before operation. A total of 10 ml anticoagulated peripheral blood was taken from each patient. The first 2 ml of blood was discarded to avoid potential contamination by normal epithelial cells, and the remaining 8 ml was collected into 20-ml sterilized tubes containing EDTA.

### Specimen processing

One ml of saline and 2 ml of lymphocyte separation medium were added to 1 ml of anticoagulated blood. Samples were centrifuged at 2500 rpm for 20 min after standing, and the white cell layer was drawn out and centrifuged at 12000 rpm for 5 min. Trizol (1 ml) was added to the precipitate, and was fully mixed. We then added 0.2 ml chloroform to the precipitate, which was mixed on a shaker at 4°C for 5 min and then centrifuged at 12000 rpm for 10 min. We collected 500 ul of the upper aqueous phase into a sterilized centrifuge tube and added an equal volume of isopropanol. The tube was gently mixed at 4°C for 10 min and then centrifuged at 12000 rpm for 15 min.

### RNA preparation

The RNA precipitate was washed with 1 ml of 75% ethanol and centrifuged at 7500 rpm for 5 min at 4°C. Total cellular RNA was extracted by the acid guanidium thiocyanate-phenol-chloroform extraction procedure [20]. The RNA was resuspended in 25 ul of diethy pyrocarbonate (DEPC)-treated water and spectrophotometrically quantitated at 260 nm and 280 nm to assess purity. Extracted RNA was treated with RNase-free Dnase I (TaKaRa Biotechnology, Da Lian, China) according to the manufacturer's instructions to remove contaminating DNA.

### Reverse transcription reaction

CK19 reverse transcription was performed according to manufacture's instructions. Briefly, 1 ug of RNA was reverse-transcribed for 15 min at 37°C in a 25 ul reaction mix containing 0.5 ul PrimeScript RT enzyme mix I, 0.5 ul oligo dT primer, 2 ul random 6-mers and 10 ul RNase free dH_2_O. The reaction was terminated by heating at 85°C for 5 sec.

### RT-PCR

CK19 primers were designed from previously published sequences [21]. The sense primer, 5′-AAGCTA ACCATGCAGAACCTCAACGACCGC-3′, and antisense primer, 5′-TTATTGGCAGGTCAGGAGAAGAGCC-3′, were used for the first round of amplification. The second PCR amplification was carried out using the CK19 sense primer, 5′-TCCCGCGACTACAGCCACTACTAC ACGACC-3′, and antisense primer, 5′-CGCGACTTGATG TCCATGAGCCGCTGGTAC-3′. PCR reaction conditions were as follows: 94°C for 3 min and 94°C for 30 sec for 40 cycles, followed by a final extension at 60°C for 35 sec. All RT-PCR products were separated by electrophoresis in a 1.5% agarose gel and were analyzed automatically via computer. CK19 levels were considered positive when copies ≥ 1000.

### Surgical tumor resection

Every patient in the study underwent mastectomy or lumpectomy and SLNB by blue dye method. If SLNs were positive, the patient underwent ALND. The number and statuses of SLNs and nSLNs were recorded, along with clinical and pathological parameters.

### Statistical analysis

In this study, all data were analyzed using SPSS (v22.0). We used χ^2^ tests (and Fisher's exact test if necessary) to analyze numerical data. The relationships between non-SLNs, clinical and pathological parameters and CK19 mRNA levels were explored by binary logistic regression analysis (enter method). ROC curve was also used for estimating the value of blood CK19 mRNA for predicting non-SLNs status pre-surgery. Significance was defined as *p* < 0.05.
